# Effects of a radiation dose reduction strategy for computed tomography in severely injured trauma patients in the emergency department: an observational study

**DOI:** 10.1186/1757-7241-19-67

**Published:** 2011-11-03

**Authors:** Soo Hyun Kim, Seung Eun Jung, Sang Hoon Oh, Kyu Nam Park, Chun Song Youn

**Affiliations:** 1 Department of Emergency Medicine, College of Medicine, The Catholic University of Korea, Seoul, Korea; 2 Department of Radiology, College of Medicine, The Catholic University of Korea, Seoul, Korea

**Keywords:** radiation dosage, computed tomography, multiple trauma

## Abstract

**Background:**

Severely injured trauma patients are exposed to clinically significant radiation doses from computed tomography (CT) imaging in the emergency department. Moreover, this radiation exposure is associated with an increased risk of cancer. The purpose of this study was to determine some effects of a radiation dose reduction strategy for CT in severely injured trauma patients in the emergency department.

**Methods:**

We implemented the radiation dose reduction strategy in May 2009. A prospective observational study design was used to collect data from patients who met the inclusion criteria during this one year study (intervention group) from May 2009 to April 2010. The prospective data were compared with data collected retrospectively for one year prior to the implementation of the radiation dose reduction strategy (control group). By comparison of the cumulative effective dose and the number of CT examinations in the two groups, we evaluated effects of a radiation dose reduction strategy. All the patients met the institutional adult trauma team activation criteria. The radiation doses calculated by the CT scanner were converted to effective doses by multiplication by a conversion coefficient.

**Results:**

A total of 118 patients were included in this study. Among them, 33 were admitted before May 2009 (control group), and 85 were admitted after May 2009 (intervention group). There were no significant differences between the two groups regarding baseline characteristics, such as injury severity and mortality. Additionally, there was no difference between the two groups in the mean number of total CT examinations per patient (4.8 vs. 4.5, respectively; p = 0.227). However, the mean effective dose of the total CT examinations per patient significantly decreased from 78.71 mSv to 29.50 mSv (p < 0.001).

**Conclusions:**

The radiation dose reduction strategy for CT in severely injured trauma patients effectively decreased the cumulative effective dose of the total CT examinations in the emergency department. But not effectively decreased the number of CT examinations.

## Background

Over the last decade, the use of computed tomography (CT) scanning has approximately doubled, and CT scanning represents approximately two thirds of the total effective radiation dose in the United States [1]. Particularly in trauma patients, CT has become an essential diagnostic tool for treatment. Accordingly, the utilization of CT has increased over time in severely injured trauma patients [2]. The biological effects of ionizing radiation have been investigated and debated for more than a century [3]. Among them, the harmful effects of ionizing radiation have been well documented. More specifically, radiation exposure has been clearly linked to the development of cancer [4].

The liberal use of CT scanning has raised concerns about issues ranging from inappropriate resource use to the consequences of radiation exposure in trauma patients. For perspective, recent studies have shown that the median cumulative effective dose of ionizing radiation can be as high as 40.2 mSv for CT scans of blunt trauma patients [5]. According to our previous study, severely injured patients were exposed to extremely high (73.8 mSv) cumulative effective doses from CT scans in the emergency department [6].

With the increasing concerns about radiation exposure with the use of CT, several groups have proposed that guidelines should be established for a more selective use of CT and low-dose radiologic CT techniques [7,8]. However, the ability of such guidelines to reduce radiation exposure has not yet been clarified. We have proposed and implemented a radiation dose reduction strategy for severely injured trauma patients. The purpose of this study was to identify the effects of a radiation dose reduction strategy for CT in severely injured trauma patients in the emergency department through a comparison of retrospective data (from before the implementation of the radiation dose reduction strategy) and prospective data (from after implementation of the strategy).

## Materials and methods

### Patients

This prospective observational study was conducted in a tertiary urban educational hospital, the Seoul St. Mary's Hospital. The study was designed to collect data from patients who met the inclusion criteria during the year-long prospective study from May 2009 to April 2010 (Intervention group). These data were compared with data collected retrospectively during the year prior to the implementation of the radiation dose reduction strategy, from May 2008 to April 2009 (Control group). This study was approved by our institutional review board. Patients whose conditions resulted in the activation of the trauma team were included in the study if they were 18 years or older and were not transferred from or to another acute care facility. Patients who did not undergo any CT scans were excluded from the study. Trauma team activation occurred when at least one physical examination item and one degree of injury item were satisfied (Additional File 1).

### Data Collection

Medical records were collected from the patients who triggered trauma team activation. The following demographic and clinical information was collected for each patient: age, sex, mechanism of injury, injury severity score (ISS), revised trauma score (RTS), Glasgow Coma Scale (GCS) score, mean arterial pressure at admission, heart rate at admission, initial disposition and outcome. The ISS was categorized as moderate injury (9-15), severe injury (16-24), or very severe injury (> = 25). The GCS score was categorized as mild injury (13-15), moderate injury (9-12), or severe injury (3-8) [9]. Established clinical definitions were used for the physiological vital signs at admission, and hypotension was defined as < 90 mmHg in systolic blood pressure. Outcomes were defined as follows: emergency department length of stay (LOS) was defined as the time from admission to departure from the emergency department, intensive care unit LOS, hospital LOS and mortality was defined as the mortality status at hospital discharge.

### CT Scanning Parameters

All the recorded CT scans conducted during admission to the emergency department were included in the study. Two 64-channel scanners (LightSpeed VCT; GE HealthCare, Milwaukee, Wisconsin, USA and Somatom Sensation 64; Siemens Medical Solutions, Erlangen, Germany) were used for all the CT studies performed before implementation of radiation dose reduction and Somatom Senstation was used after implementation of radiation dose reduction. The general scan parameters for each trauma location before the implementation of dose reduction strategy were a) head trauma: helical acquisition, 120 kVp, 400 mA, 112 effective mAs, 1, 46 sec of rotation time, 3.8 mm slice thickness, and standard of convolutional kernel for LightSpeed VCT and 120 kVp, 390 effective mAs, 1 sec of rotation time, 5 mm slice width, and H31s of convolutional kernel to spiral mode; b) spine: helical acquisition, 120 kVp, 335 mA, 8 effective mAs, 1, 46 sec of rotation time, 2 mm slice thickness, and standard of convolutional kernel for LightSpeed VCT; (c) chest: Helical acquisition, 120 kVp, 250 mA, 15 effective mAs, 0.5 sec of rotation time, 5-mm slice thickness and high resolution kernel for lung window setting for non-enhanced scan and standard convolutional kernel for mediastinal setting on contrast enhanced scan. Two phases of before and after contrast administration were obtained; (d) abdomen and pelvis: helical acquisition, 120 kVp, 304 mA, automatic tube current modulation (SmartmA), 0.5 sec of rotation time, 5-mm slice thickness and standard convolutional kernel. Two phases of arterial phase and portal venous phase after contrast administration with bolus tracking were obtained. When we obtained both chest CT and abdominopelvic CT, we obtained each body parts scanning according to each protocol. The upper abdomen was overlapped in chest CT and abdominopelvic CT.

After implementation of dose reduction strategy, a) head trauma: helical acquisition, 120 kVp, 390 effective mAs, 0.5 sec of rotation time, 5 mm slice width, and H31s of convolutional kernel; b) spine: helical acquisition, 120 kVp, 180 mAs, 0.5 sec of rotation time, 0.6 mm slice thickness; (c) chest, abdomen and pelvis: Helical acquisition, 120 kVp (100 kVp in cases of patients with body weight under 50 kg for woman and 60 kg for man), 130 reference mAs with automatic tube current modulation (CARE Dose 4D), 0.5 sec of rotation time, and 5-mm slice thickness. We used soft kernel of B30f for abdomen and pelvis. For chest, two phases of non-enhanced and enhanced scan. For abdomen and pelvis, only one phase after contrast administration was obtained. Bolus tracking was not applying for the contrast enhanced CT. Fixed contrast enhancement delay time (80 sec) was used. For chest, abdomen and pelvis, first, non-enhanced chest CT was obtained and then contrast enhanced scan with scan range from thoracic inlet to pelvic cavity was obtained.

The frequency of CT examinations counted the times the patients have been driven through the scanner by each part. The mean number of CT examinations per patient was recorded, as was any change in the mean number of CT examinations per patient after stratifying by patient demographics. Estimates of the cumulative effective dose (CED) of radiation were determined by converting the dose length product (DLP) for the CT examinations to the effective dose using the formula E = E_DLP _× DLP, where E is the CED (mSv), E_DLP _is the normalized effective dose (mSv mGy^-1 ^cm^-1^), and DLP is the dose length product per CT scan. The DLP for each CT examination was displayed as an image after the completion of the scan. The E_DLP _values for the head, neck, chest, abdomen, pelvis, and extremities were referenced from the European Guidelines on Quality Criteria for Computed Tomography [7]. The E_DLP _values for contiguous scans, including head/neck scans and chest/abdomen/pelvic scans, were calculated as needed. A mean CED value was calculated for each procedure.

### Radiation Dose Reduction Strategy

Beginning in May 2009, we implemented a radiation dose reduction strategy, which included two major components. One approach involved radiologic technical efforts using a low-dose radiation CT protocol, which consisted of reducing the tube voltage in patients with low body weight, employing automatic tube current modulation, using 1-phase CT instead of 2-phases, using soft convolutional kernel and removing overlapping scan. The other approach involved reducing the number of CT scanning without scarifying information about injured patients. This approach included reducing repeated CT scanning, using alternative imaging methods, such as ultrasonography and magnetic resonance imaging, and returning to an increased reliance on clinical examinations.

### Statistical Analyses

Categorical data are expressed as frequencies and percentages. Numerical data are expressed as means and 95% confidence interval. The data were assessed for normal distribution using the Kolmogorov-Smirnov test. Chi-squared tests were used to determine differences in patient demographic data between the two groups. Student's t tests were used to identify differences in CT examination frequency and CED. All the statistical analyses were performed using SPSS software, version 17.0. Values of p < 0.05 were considered statistically significant for all comparisons.

## Results

### 1. Patient Demographics

Over a 2-year period, 132 patients triggered full activation of the trauma team. Six patients who were transferred to our trauma center from outside facilities with prior imaging did not require further CT scans. Five patients were children, and 3 of the patients died without a CT evaluation, which could not be performed because of unstable hemodynamics. Thus, the analysis included 118 subjects with complete data. Among these 118 patients, 33 were admitted before May 2009 (control group), and 85 were admitted after May 2009 (intervention group). Baseline characteristics are presented in Table 1. There were no significant differences in age, gender, or trauma mechanism between the two groups. The majority of the patients included in the study were male (72.7% and 72.9% for the two groups, respectively) and suffered blunt injuries (84.8% and 91.8%, respectively). Also there were no significant differences between the two groups in any of parameters presenting injury severity, such as ISSs, RTSs and head injury. Hemodynamic status between the two groups was not showed significant differences.

**Table 1:** Patient demographics

	Control group (n = 33)	Intervention group (n = 85)	p
Age (yrs)	41.9 (36.1-47.6)	47.1 (43.8-51.1)	0.131
Male (n, %)	24 (72.7)	62 (72.9)	0.981
Mechanism (n, %)			0.265
Blunt	28 (84.8)	78 (91.8)	
Penetrating	5 (15.2)	7 (8.2)	
ISS	22.8 (19.2-26.5)	25.2 (22.6-27.9)	0.330
ISS severity (n, %)			0.533
Moderate (9-15)	7 (21.2)	11 (12.9)	
Severe (16-24)	12 (36.4)	34 (40.0)	
Very severe (≥ 25)	14 (42.4)	40 (47.1)	
RTS	6.9 (6.5-7.3)	6.5 (6.2-6.9)	0.138
Head injury (n, %)			0.366
Mild (GCS 13-15)	21 (63.6)	43 (50.6)	
Moderate (GCS 9-12)	4 (12.1)	10 (11.8)	
Severe (GCS 3-8)	8 (24.2)	32 (37.6)	
Hemodynamic status			
MAP (mmHg)	85.9(78.4-93.3)	92.7(85.4-99.5)	0.182
HR (beats/min)	92.2(84.6-99.7)	88.6(82.2-95.8)	0.544
Shock (n, %)	5 (15.2)	12 (14.1)	0.886
Initial disposition (n, %)			0.962
Operation	13 (39.4)	29 (34.1)	
Admission	9 (27.3)	25 (29.4)	
Transfer	7 (21.2)	20 (23.5)	
Death	4 (12.1)	11 (12.9)	
ED LOS (hours)	14.1 (9.2-19.1)	14.3 (8.9-19.8)	0.962
ICU LOS (days)	4.6 (2.7-6.4)	5.6 (3.7-7.5)	0.512
Hospital LOS (days)	26.2 (17.8-34.5)	31.0 (22.7-39.3)	0.495
Mortality (n, %)	7 (21.2)	14 (16.5)	0.546

Data are means (95% confidence interval) unless noted.

ISS: injury severity score; RTS: revised trauma score; GCS: Glasgow Coma Scale; MAP: mean arterial pressure; HR: heart rate; ED LOS: emergency department length of stay; ICU: intensive care unit

### 2. Patient Outcomes

There were no significant differences between the two groups in initial disposition, emergency department length of stay and mortality (Table 1). For the two groups, 13 (39.4%) and 29 patients (34.1%), respectively, received emergency surgery; 9 (27.3%) and 25 patients (29.4%), respectively, were hospitalized; and 7 (21.2%) and 20 patients (23.5%), respectively, were transferred to other hospitals for long-term in-patient care. The mean intensive care unit length of stay was 4.6 days for control group and 5.6 days for intervention group (p = 0.512). For the two groups, there were 7 (21.2%) and 14 deaths (16.5%), respectively, during the study period. Of these deaths, 4 and 11 occurred in the emergency room for the two groups, respectively.

### 3. Cumulative Effective Dose

**Figure 1:**
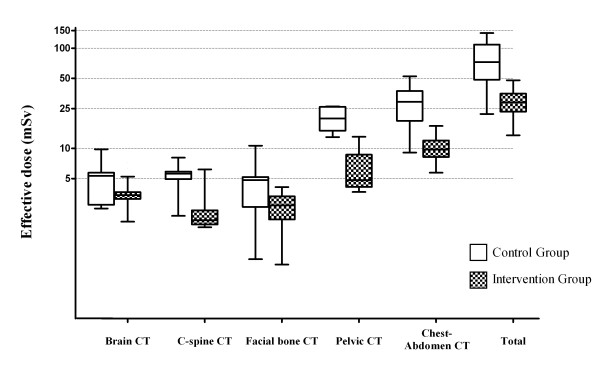
**Box and whisker plot with median, 25th and 75th percentiles for the effective radiation dose from CT for the two groups**. Control group included patients from May 2008 to April 2009, before use of the dose reduction strategy, and intervention group included patients from May 2009 to April 2010, after implementation of the dose reduction strategy.

The cumulative effective dose of radiation administered to each patient during the study periods, decreased significantly from 78.71 mSv (control group) to 29.50 mSv (intervention group) per patient (p < 0.001). The mean CED per examination by anatomical region is presented in Table 2. Notably, the mean brain CT CED decreased from 4.88 mSv to 3.27 mSv, and the mean CED for a chest/abdomen CT decreased from 28.42 mSv to 9.91 mSv (Figure 1). More specifically, this corresponded to significant decreases of 33% and 65%, respectively (p < 0.001).

**Table 2:** Mean cumulative effective doses (mSv)

	Control group	Intervention group	p
Brain CT	4.88 (4.10-5.65)	3.27 (3.01-3.51)	< 0.001
Cervical spine CT	1.31 (0.43-2.17)	1.18 (0.85-1.50)	0.779
Facial bone CT	2.88 (1.90-3.84)	1.03 (0.73-1.35)	0.001
Pelvic bone CT	3.66 (0.76-6.55)	0.75 (0.25-1.26)	0.053
Chest-Abdomen CT	28.42 (24.15-32.67)	9.92 (9.17-10.76)	< 0.001
Total CT	78.71 (65.80-91.61)	29.50 (27.37-31.74)	< 0.001

Data are means (95% confidence interval) unless noted.

CT: Computed tomography

### 4. CT Examination Frequency

During the study periods, the mean number of CT examinations per patient was 4.8 times and 4.5 times (p = 0.227) for the two groups, respectively, which was not a significant difference. Likewise, there was no significant difference between the two groups in the total frequency of CT examinations by demographic variables. Compared with the control group, the mean number of CT examinations per patient was significantly lower in intervention group patients with ISSs of 8-15 (p = 0.028) and those with mild head injuries (p = 0.020) (Table 3).

**Table 3:** Frequency of total CT scans by demographic variables

	Control group	Intervention group	p
Male	4.8 (4.2-5.3)	4.6 (4.2-4.9)	0.562
Female	5.0 (3.8-6.2)	4.3 (3.8-4.8)	0.157
Age ≥ 65 yrs	4.9 (4.3-5.4)	4.5 (4.2-4.8)	0.279
Age < 65 yrs	4.5 (1.9-10.8)	4.4 (3.7-4.9)	0.850
Blunt mechanism	5.0 (4.5-5.5)	4.3 (3.7-5.0)	0.186
Penetrating mechanism	3.8 (1.9-5.6)	3.0 (1.4-4.5)	0.400
ISS severity			
Moderate (9-15)	5.3 (4.41-6.17)	3.9 (2.88-4.76)	0.028
Severe (16-24)	5.0 (3.9-5.9)	4.9 (4.4-5.3)	0.896
Very severe (≥ 25)	4.5 (3.6-5.4)	4.4 (3.9-4.7)	0.680
Head injury			
Mild (GCS 13-15)	5.0 (4.3-5.6)	4.1 (3.6-4.5)	0.020
Moderate (GCS 9-12)	4.8 (1.5-8.0)	5.0 (3.8-6.2)	0.818
Severe (GCS 3-8)	4.5 (3.4-5.6)	4.9 (4.5-5.3)	0.391
Shock	4.0 (3.1-4.9)	5.0 (4.2-5.6)	0.105
Total frequency of CT	4.8 (4.3-5.3)	4.5 (4.2-4.8)	0.227

Data are means (95% confidence interval) unless noted.

ISS: injury severity score; GCS: Glasgow Coma Scale; CT: Computed tomography

## Discussion

The average annual radiation exposure per person from all environmental sources varies from 2.2 to 3.6 mSv [10]. Ionizing radiation is the most studied ubiquitous carcinogen in our environment [11]. The biological effects of low-dose ionizing radiation have been investigated and debated for more than a century. The most recent comprehensive assessment of the health risks from exposure to low levels of ionizing radiation is the Biological Effects of Ionizing Radiation (BEIR) VII Phase 2 report, which was published in 2006 by the National Research Council of the National Academy of Sciences. This report states that of 100, 000 people exposed to a dose of 100 mSv, an additional 800 cases of cancer are predicted. This risk is linear, suggesting that the mean dose of 68.8 mSv observed in our previous study would contribute to an additional 551 cancer cases per 100, 000 subjects exposed [6,12]. The Radiation Effects and Research Foundation has extensively studied the effects of radiation on atomic bomb survivors in Nagasaki and Hiroshima. This cohort includes 86, 572 people with individual dose estimates, 60% of whom have doses of at least 5 mSv. There have been 9, 335 deaths from solid cancer and 31, 881 deaths from noncancer diseases during the 47-year follow-up. Among them, about 440 (5%) of the solid cancer deaths and 250 (0.8%) of the noncancer deaths were associated with the radiation exposure. And there was a useful representative value that the solid cancer risk for those exposed at age 30 is elevated by 47% per sievert at age 70. The radiation doses (5-100 mSv) to which Japanese atomic bomb survivors were exposed are similar to those of CT scans, and both entail an increased risk of cancer induction and cancer-related mortality [13]. The trend toward an increasing use of CT scans could have serious public health implications. The resultant attributable risk of cancer from CT scans might be as high as 2.0% of all cancers [14].

CT scanning has an established role in the evaluation of trauma patients. Refinements in CT technology have led to excellent imaging for the diagnosis of many traumatic injuries, including solid organ injuries, spine fractures, and blunt aortic injuries. There has been a 20-fold increase in the use of CT scans in the United States over the past two decades [15]. In one study that compared mean CT examinations and utilization for trauma patients in 2003 and 2007, an overall increase in the mean number of CT examinations per patient was observed in 2007 (4.41 in 2007 vs. 3.44 in 2003; p = 0.002) [16]. This increase entailed a significant overall rise in ionizing radiation exposure and its associated risks, particularly carcinogenesis. As a result, in a 2004 study on whether this increased use of CT is indeed helpful, Fenton et al. [17] suggested that, especially in pediatric blunt trauma patients, emergency room professionals may be "overdoing it" with CT scans. In that retrospective review, 1, 422 children received 2, 361 scans, or 1.7 scans per patient, and 54% of the scans were interpreted as normal. Medical providers should be aware of the increased cancer risk associated with radiation when ordering CT studies for patients with trauma.

In a 2004 study by Kim et al. [18] that quantitatively analyzed the CED for trauma patients, the CED from all radiologic studies in critically ill patients with trauma was calculated. They reported a mean CED of 106 mSv and a mean CT examination count per patient of nearly eight. The authors limited the population to critically ill patients with trauma with an ICU length of stay (LOS) > 30 days. At our level I trauma center, only 1.58% of critically ill patients (defined here as patients with ISSs > 24) had an ICU LOS > 30 days; therefore, the study population criteria and reported mean effective dose of radiation may not be generalizable to a critically ill trauma population. In one recent report, the CED was 16 mSv per patient in the first 3 hours of hospitalization, whereas the CED in the first 24 hours was 40.2 mSv in severely injured blunt trauma patients (median age: 32 years, ISS: 14) [19]. According to our previous data for a study of 33 patients with trauma team activation, the CED exposure during the initial 24 hours was also high at 73.8 mSv [6]. In 2006, Ott et al. [10] examined the radiation doses of patients at a level I trauma center through the use of dosimeter badges attached to the wrist. They found that many patients in the high-risk groups received > 1 mSv of radiation, which is the limit set by the US Nuclear Regulatory Commission for the public. However, medical facilities are exempt from these regulations because there are clear medical benefits of delivering doses of radiation > 1 mSv.

In recent years, there have been many studies on how to reduce radiation dose. A common practice to reduce radiation risk is to adjust the scan parameters, including tube current, slice spacing, and slice thickness, to reduce radiation exposure without compromising image quality. Radiation may also be reduced by following published guidelines, such as the European Guidelines on Quality Criteria for Computed Tomography or the American College of Radiology appropriateness criteria. Hadley et al. [19] reported that CT examinations performed according to the American College of Radiology guidelines would reduce radiation doses by 44% and imaging costs by 39%. Magnetic resonance imaging has also been suggested as an alternative to CT to avoid the risks associated with radiation [20]. The present study employed a uniform methodology for CT imaging to reduce the effective dose. As a result, the total CED exposure of each patient decreased from 73.8 mSv (range, 65.8-91.6) to 29.5 mSv (range, 27.3-31.7). An analysis showed that the reduction in CED from chest/abdomen CT scans played a major role in this large decrease in the amount of CED, which was the result of multiple efforts, including shortening the radiation exposure time by reducing the CT scan phase from 2 to 1 and adjusting the hand position. Additionally, 10 patients in this study had radiation exposures over 100 mSv, and all ten belonged to control group, which indicates that the guidelines for CED reduction were effective.

Mueller et al. [21] showed that the selective scanning of body areas, as compared with whole body scanning, resulted in significant decreases in all organ doses and the total effective dose; moreover, this study concluded that the development of clinical decision rules is necessary. In our study, after establishing CT scan guidelines according to clinical symptoms and comparing the number of CT scans before and after the implementation of these guidelines, we found that there was no difference in the total number of CT scans per patient between the two groups. It is because that the severity of the patient was similar between the two groups and that the patients included in our study were severely injured with trauma, so it has not been able to reduce the number of CT required for diagnosis. However, an examination of the number of CT scans for both groups according to each variable revealed significant reductions in the number of CT scans for the moderate ISS (8-15) group and the group with mild head injuries (GCS 13-15). This result may indicate that unnecessary CT scans could be reduced for patients with mild loss of consciousness and replaced by more accurate physical examinations, which indicates that the number of CT evaluations can be adjusted depending on the degree of injury severity in each patient. Other general methods of reducing radiation exposure include limiting the use of fluoroscopy during procedures, increasing the use of lead shielding for areas not included in the examination, and changing protocols to assess testing under routine and non-urgent situations [10]. Additionally, alternative, non-ionizing modalities, such as ultrasonography or magnetic resonance imaging, could be considered for severely injured trauma patients.

## Limitations

The first limitation of this study is that the CED was not directly measured but rather was an estimate of the radiation dose given to the entire body. Second, the whole-body CEDs of all the patients were used because the relationship between the severity classification according to the patient's clinical presentation and the CT for each area could not be analyzed. Third, differences in patient outcome could not be considered because there was no comparison of CT image quality. To solve these problems, patients who are discharged from the emergency department need to receive follow-up process whether delayed or missed injury or not. And it would be needed additional processes that surgeons and radiologists evaluate the quality of imaging. Forth, there was difference in the number of included patient between the two groups due to our institution's own problem. Finally, the number of samples was small because the study was performed in a local university hospital and because a clear analysis of the final outcomes of patients transferred to other hospitals could not be completed.

## Conclusion

The radiation dose reduction strategy for CT significantly decreased the cumulative effective dose of total CT scans, especially abdominal CT scans, in severely injured trauma patients in the emergency department. However, the frequency of CT scans was not significantly decreased despite the use of this strategy.

## Abbreviations

CT: computed tomography; ISS: injury severity score; RTS: revised trauma score; GCS: Glasgow Coma Scale; CED: cumulative effective dose; ICU: intensive care unit; LOS: length of stay.

## Competing interests

The authors declare that they have no competing interests.

## Authors' contributions

SHK performed data analysis and drafted the manuscript. SEJ acquired data and critical revisions to the manuscript. SHO and KNP managed the data and critical revisions to the manuscript. CSY conceived the research and drafted the manuscript. Each authors has read and approved the final manuscript.

## Supplementary Material

Additional file 1 **The criteria for trauma team activation**. Click here for file
