# Supplementary material for: Effects of a radiation dose reduction strategy for computed tomography in severely injured trauma patients in the emergency department: an observational study
Source: Scand J Trauma Resusc Emerg Med. 2011 Nov 3;19:67. doi: 10.1186/1757-7241-19-67 (PMC3231876; doi:10.1186/1757-7241-19-67)
Supplement: Additional file 1 — The criteria for trauma team activation. [file 1757-7241-19-67-S1.DOC]

**Additional File 1**

| Physical Examination |
| --- |
| Trauma arrest  Systolic BP <90 mmHg  Decreased mentality  Loss of pupillary light reflex  Airway problem (intubated or attempted intubation) |
| Injuries |
| Flail chest  Proximal long bone Fracture (more than 2)  Spinal injury with paralysis  Stab wound (head, chest, abdomen, pelvis)  Proximal amputation or crush injury  Skull fracture (open or FCCD) |
